# Large-Scale Neuronal Network Dysfunction in Diabetic Retinopathy

**DOI:** 10.1155/2020/6872508

**Published:** 2020-01-22

**Authors:** Xin Huang, Yan Tong, Chen-Xing Qi, Han-Dong Dan, Qin-Qin Deng, Yin Shen

**Affiliations:** ^1^Eye Center, Renmin Hospital of Wuhan University, Wuhan, 430060 Hubei, China; ^2^Medical Research Institute, Wuhan University, Wuhan, 430071 Hubei, China

## Abstract

Diabetic retinopathy (DR) patients are at an increased risk of cognitive decline and dementia. There is accumulating evidence that specific functional and structural architecture changes in the brain are related to cognitive impairment in DR patients. However, little is known regarding whether the functional architecture of resting-state networks (RSNs) changes in DR patients. The purpose of this study was to investigate the intranetwork functional connectivity (FC) and functional network connectivity (FNC) of RSN changes in DR patients using independent component analysis (ICA). Thirty-four DR patients (18 men and 16 women; mean age, 53.53 ± 8.67 years) and 38 nondiabetic healthy controls (HCs) (15 men and 23 women; mean age, 48.63 ± 11.83 years), closely matched for age, sex, and education, underwent resting-state magnetic resonance imaging scans. ICA was applied to extract the nine RSNs. Then, two-sample *t*-tests were conducted to investigate different intranetwork FCs within nine RSNs between the two groups. The FNC toolbox was used to assess interactions among RSNs. Pearson correlation analysis was conducted to explore the relationship between intranetwork FCs and clinical variables in the DR group. A receiver operating characteristic (ROC) curve was conducted to assess the ability of the intranetwork FCs of RSNs in discriminating between the two groups. Compared to the HC group, DR patients showed significant decreased intranetwork FCs within the basal ganglia network (BGN), visual network (VN), ventral default mode network (vDMN), right executive control network (rECN), salience network (SN), left executive control network (lECN), auditory network (AN), and dorsal default mode network (dDMN). In addition, FNC analysis showed increased VN-BGN, VN-vDMN, VN-dDMN, vDMN-lECN, SN-BGN, lECN-dDMN, and AN-BGN FNCs in the DR group, relative to the HC group. Furthermore, altered intranetwork FCs of RSNs were significantly correlated with the glycosylated hemoglobin (HbA1c) level in DR patients. A ROC curve showed that these specific intranetwork FCs of RSNs discriminated between the two groups with a high degree of sensitivity and specificity. Our study highlighted that DR patients had widespread deficits in both low-level perceptual and higher-order cognitive networks. Our results offer important insights into the neural mechanisms of visual loss and cognitive decline in DR patients.

## 1. Introduction

Diabetic retinopathy (DR) is a serious diabetic retinal microvascular complication and one of the major causes of blindness worldwide [1]. The global prevalence of DR is reportedly 34.6% among diabetes patients [2]. There are several risk factors for DR, such as a higher hemoglobin (HbA1c) level [3], longer duration of diabetes [4], and hypertension [5]. The main pathological changes in DR are capillary nonperfusion, vascular leakage, and retinal neurodegeneration. These are followed by proliferative retinal detachment and eventual blindness. Importantly, the retinal vasculature shares similar anatomic, physiological, and embryological characteristics to cerebral vessels [6]. There is growing evidence that DR patients are at high risk of stroke [7] and cerebral microbleeds [8]. Furthermore, DR has been associated with cognitive decline [9, 10]. Naidu et al. reported that increased retinal venular tortuosity was related to cognitive decline in patients with type 2 diabetes mellitus (T2DM) [11]. However, the etiology of the neurophysiological mechanism of this increased risk is unclear.

Recent advances in magnetic resonance imaging approaches have provided a powerful framework for the characterization of central nervous system changes in DR. Previous neuroimaging studies demonstrated that specific functional and structural architecture changes in the brain are related to cognitive impairment in DR patients. Wang et al. demonstrated that increased amplitude of low-frequency fluctuation (ALFF) values in the right occipital lobe was correlated with cognitive impairments in DR patients [12]. van Duinkerken et al. reported that type 1 diabetes patients with proliferative retinopathy showed lower eigenvector centrality mapping and degree centrality in the bilateral thalamus and dorsal striatum, as well as increased eigenvector centrality mapping in the occipital cortex [13]. Dai et al. demonstrated that DR patients had abnormal brain local functional networks related to visual function and cognition [14]. DR patients also exhibit brain structural alterations. Wessels et al. demonstrated that decreased gray matter density in the right inferior frontal gyrus and right occipital lobe was observed in DR patients, relative to healthy controls (HCs) [15]. Moreover, van Duinkerken et al. reported that type 1 diabetes mellitus patients with proliferative retinopathy showed lower local clustering in the middle frontal, postcentral, and occipital areas [16]. DR patients showed decreased putamen and right thalamic volume relative to HCs [17]. The abovementioned studies mainly focused on local functional and structural changes in the brains of DR patients. However, it is largely unknown how large-scale neuronal networks are affected by DR.

Low-frequency fluctuations (<0.01 Hz) in the blood oxygenation level-dependent signal during rest reflect spontaneous neural activity, which can be conceptualized as a network of anatomically linked regions. Low-level perceptual and higher-order cognitive networks engage in organized patterns of correlated activity forming resting-state networks (RSNs) [18–20]. Previous neuroimaging studies demonstrated that RSNs can be divided into perceptual networks (visual, sensorimotor, and auditory), higher-order cognitive networks (default mode, executive, and salience), and other subcortical networks (basal ganglia network (BGN)) [21, 22]. Independent component analysis (ICA) is a powerful data-driven approach for finding independent patterns in multivariate data; the ICA method is used to identify multiple RSNs and investigate intra- and internetwork functional connectivities (FCs) *in vivo* [23, 24]. Prior neuroimaging studies revealed that widespread abnormal RSNs were observed in diabetic patients. van Duinkerken et al. reported that type 1 diabetes mellitus patients with microangiopathy showed decreased intranetwork FCs of RSNs involving attention, working memory, auditory and language processing, and motor and visual processes [25]. Chen et al. demonstrated that abnormal RSNs of the default mode, left frontal parietal, and sensorimotor networks were detected in T2DM patients [26]. However, the ICA method has not been applied to characterize changes in RSNs in DR patients. There is growing evidence that DR patients exhibit greater cognitive impairment than those with advanced DR. In addition, retinal microvascular abnormalities constitute useful clinical biomarkers for cognitive decline in patients with diabetes [27]. Thus, we hypothesized that DR patients would exhibit distinct patterns of changes in RSNs, relative to diabetes mellitus patients without retinopathy.

Based on this hypothesis, the purpose of this study was to determine whether DR patients exhibited intranetwork FCs and functional network connectivities (FNCs) associated with changes in RSNs. We investigated relationships between distinct intranetwork FCs in brain regions and clinical variables (visual function and biochemical examination) in the DR group. Our findings might provide new insights into underlying neural mechanisms in DR.

## 2. Materials and Methods

### 2.1. Participants

Thirty-four DR patients (18 men and 16 women; mean age, 53.53 ± 8.67 years) and 38 nondiabetic HCs (15 men and 23 women; mean age, 48.63 ± 11.83 years), matched for age, sex, and education, participated in this study. All participants enrolled in the study met the following criteria: (1) they had no cardiac pacemaker or implanted metal devices and could undergo magnetic resonance imaging; (2) they did not have heart disease or claustrophobia; and (3) they did not have cerebral diseases, as determined by high-resolution T1-weighted images assessed by an experienced radiologist.

The diagnostic criteria of DR patients were (1) fasting plasma glucose ≥ 7.0187mmol/L, random plasma glucose ≥ 11.1 mmol/L, or 2‐hour glucose ≥ 11.1 mmol/L and (2) patients with nonproliferative DR exhibiting microaneurysms, hard exudates, and retinal hemorrhages. The exclusion criteria for DR patients were (1) the presence of proliferative DR with retinal detachment; (2) the presence of vitreous hemorrhage; (3) the presence of additional ocular-related complications (cataract, glaucoma, high myopia, or optic neuritis); and (4) the presence of diabetic nephropathy or diabetic neuropathy.

All HCs met the following criteria: (1) fasting plasma glucose < 7.0 mmol/L, random plasma glucose < 11.1 mmol/L, and HbA1c < 6.5%; (2) no ocular diseases (myopia, cataracts, glaucoma, optic neuritis, or retinal degeneration); (3) binocular visual acuity ≥ 1.0 (decimal); (4) no ocular surgical history; and (4) no mental disorders.

### 2.2. Ethical Statement

The research protocol adhered to the tenets of the Declaration of Helsinki and was approved by the institutional review board of Renmin Hospital of Wuhan University. All subjects provided written informed consent to participate in the study.

### 2.3. MRI Acquisition

MRI scanning was performed on a 3-tesla magnetic resonance scanner (Discovery MR750W system; GE Healthcare, Milwaukee, WI, USA) with an eight-channel head coil. Whole-brain T1 weights were obtained with three-dimensional brain volume imaging (3D-BRAVO) MRI with the following parameters: repetition time (TR)/echo time (TE) = 8.5/3.3, thickness = 1.0 mm, no intersection gap, acquisition matrix = 256 × 256, field of view = 240 × 240 mm^2^, and flip angle = 12°.

Functional images were obtained by using a gradient echoplanar imaging sequence with the following parameters: TR/TE = 2,000 ms/25 ms, thickness = 3.0 mm, gap = 1.2 mm, acquisition matrix = 64 × 64, flip angle = 90°, field of view = 240 × 240 mm^2^, voxel size = 3.6 × 3.6 × 3.6 mm^3^, and 35 axial slices. All the subjects were instructed to rest quietly with their eyes closed and relaxed without thinking about anything in particular or falling asleep.

### 2.4. Data Analysis

All preprocessing was performed using the toolbox for Data Processing & Analysis of Brain Imaging (DPABI, http://www.rfmri.org/dpabi) [28] which is based on Statistical Parametric Mapping (SPM12) (http://www.fil.ion.ucl.ac.uk) implemented in MATLAB 2013a (MathWorks, Natick, MA, USA) and briefly the following steps according to our previous study [29]: (1) DICOM format of the functional images was converted to NIFTI format, and the first ten volumes of each subject were removed due to the signal reaching equilibrium. (2) The remaining 230 volumes of functional BOLD images were corrected for slice timing effects, motion corrected. For head motion parameters, more than 2 mm or for whom rotation exceeded 1.5° during scanning was excluded [30]. (3) Individual 3D-BRAVO images were registered to the mean fMRI data; then, resulting aligned T1-weighted images were segmented using the Diffeomorphic Anatomical Registration Through Exponentiated Lie Algebra (DARTEL) toolbox for improving spatial precision in the normalization of fMRI data [31]. Normalized data (in Montreal Neurological Institute (MNI) 152 space) were resliced at a resolution of 3 × 3 × 3 mm^3^. (4) Spatial smoothing is employed by convolution with an isotropic Gaussian kernel of 6 × 6 × 6 mm full width at half maximum.

### 2.5. Group ICA Analysis and Identification of RSNs

Group ICA was performed to decompose the data into independent components (ICs) using the GIFT toolbox (http://icatb.sourceforge.net/, version 3.0b) [32]. First, we estimated the dimensions of the datasets from the two groups using the minimum description length criterion to adjust for spatial correlation [33]. 26 IC maps were estimated in this study. Second, all data from each subject were reduced; the compressed datasets of each subject were concatenated into a single group, and this aggregate dataset was further reduced to 26 components using principal component analysis, followed by IC estimation using the Infomax algorithm [34]. This step was repeated 100 times using the ICASSO algorithm to assess the repeatability or stability of ICs [35]. Third, the ICs for each subject were derived from the group ICA back-reconstruction step and were converted into *z*-scores [36]. Components retained for further analysis among the 26 estimated ICs were selected based on the largest spatial correlation with specific RSN templates [37, 38]. The IC time courses and spatial maps for each subject were transformed to *z*-scores. We selected 26 meaningful ICs by using the following criteria: (a) peak coordinates of spatial maps located primarily in the gray matter, (b) no spatial overlap with vascular, ventricular, or susceptibility artifacts, and (c) time courses dominated by low-frequency signals (ratio of powers below 0.1 Hz to 0.15-0.25 Hz in the frequency spectrum). Nine RSNs were identified in this study: basal ganglia network (BGN), visual network (VN), ventral default mode network (vDMN), right executive control network (rECN), salience network (SN), left executive control network (lECN), auditory network (AN), sensorimotor network (SMN), and dorsal default mode network (dDMN).

### 2.6. Statistical Analysis

For spatial maps for each of the RSNs, the ICs corresponding to nine RSNs were extracted from all subjects and one-sample *t*-tests were performed for the spatial maps of each RSN by using SPM12 software. Statistical significance thresholds were set at *P* < 0.001 (false discovery rate- (FDR-) corrected). The group spatial maps of RSN were visualized using the Resting-State fMRI Data Analysis Toolkit plus V1.2 (RESTplus V1.2, http://restfmri.net/forum/RESTplusV1.2).

For intranetwork functional connectivity analysis, two-sample *t*-tests were used to compare differences between the two groups in the intranetwork FC within RSN maps; the Gaussian random field method was used to correct for multiple comparisons and regressed covariates of age and sex using SPM12 software. Group comparisons were masked to the voxels within corresponding RSNs (two-tailed, voxel-level *P* < 0.01; Gaussian random field correction, cluster-level *P* < 0.05). The mask was created by combining the regions of corresponding RSNs in both DR patients and HCs, which were obtained from one-sample *t*-test results. These results were shown using BrainNet Viewer software (https://www.nitrc.org/projects/bnv/).

For internetwork functional connectivity analysis, the FNC toolbox (http://trendscenter.org/software/, version 2.3) was used to calculate temporal relationships between RSNs. Corresponding to the significant correlation combinations, the average time lags were calculated for each group; these represented the amount of delay between time courses of two correlated RSNs. One-sample *t*-tests were used to compare temporal relationships between RSNs for each group (*P* < 0.05, uncorrected). Two-sample *t*-tests were used to compare distinct temporal relationships between RSNs between the two groups (*P* < 0.05, uncorrected).

### 2.7. Correlation Analyses

A Pearson correlation coefficient was conducted to assess the relationships between the intranetwork FC values of different brain regions and clinical variables in the DR group using SPSS version 20.0 software (SPSS Inc., Chicago, IL, USA).

## 3. Results

### 3.1. Demographics and Visual Measurements

There was significant difference in BCVA-OD (*P* < 0.001) and BCVA-OS (*P* < 0.001). There were no significant differences in the gender, age, and weight between the groups (Table 1).

### 3.2. Spatial Pattern of RSNs in Each Group

**Figure 1:**
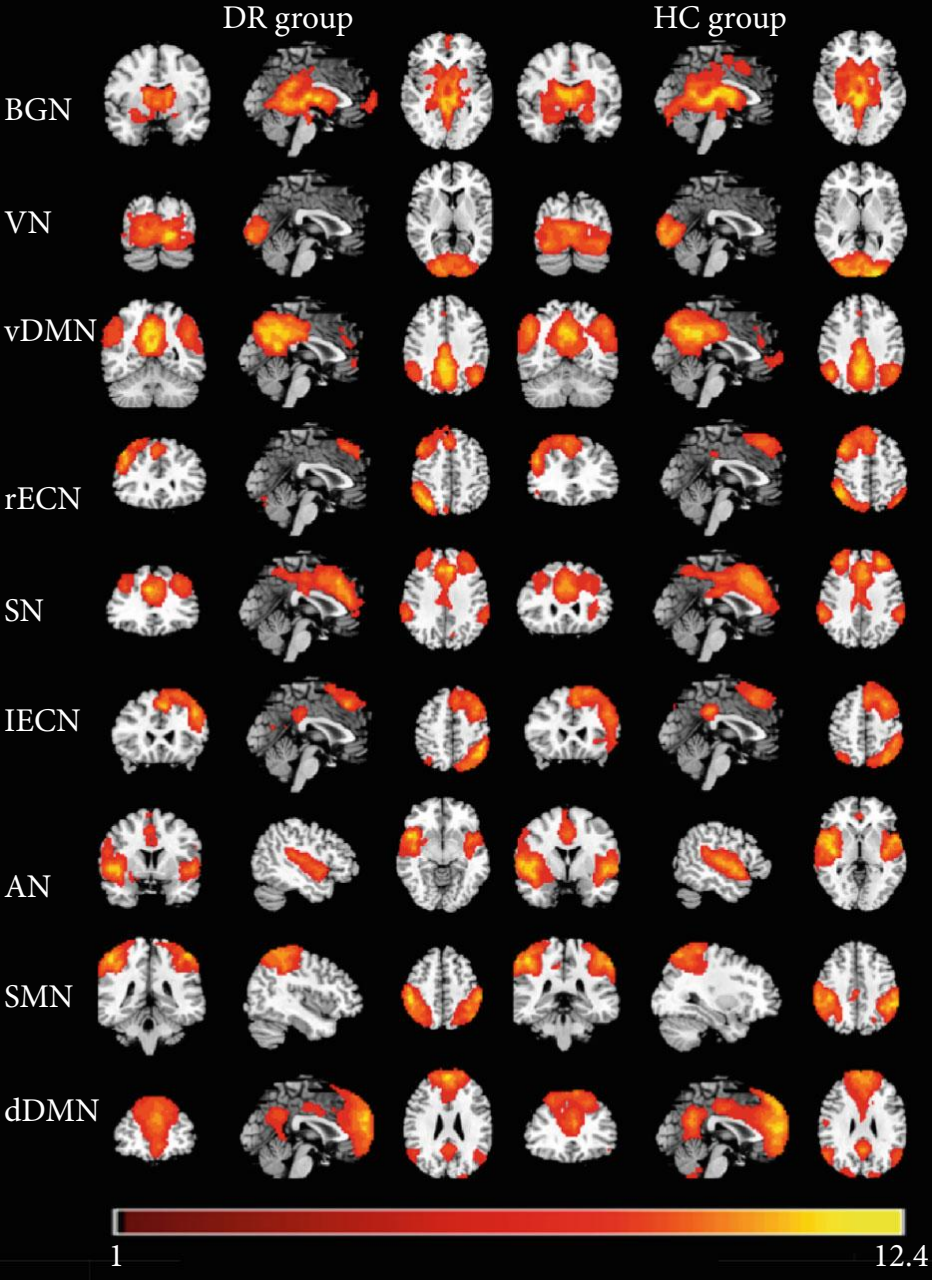
The typical spatial patterns in each RSN of both DR and HC groups, including BGN, VN, vDMN, rECN, SN, lECN, AN, SMN, and dDMN. Scale represents *t* values with a range of 1~12.4 in each RSN (*P* < 0.001, FDR corrected). Abbreviations: DR: diabetic retinopathy; HC: healthy control; BGN: basal ganglia network; VN: visual network; vDMN: ventral default mode network; rECN: right executive control network; SN: salience network; lECN: left executive control network; AN: auditory network; SMN: sensorimotor network; dDMN: dorsal default mode network; RSN: resting-state networks; FDR: false discovery rate.

The typical spatial patterns in each RSN of both DR and HC groups are illustrated in Figure 1. Nine of these components coincided with RSNs included: (1) basal ganglia network (BGN): putamen, caudate nucleus, pallidum, substantia nigra, and subthalamic nucleus; (2) visual network (VN): middle occipital gyrus, superior occipital gyrus, the temporal-occipital regions, and fusiform gyrus; (3) ventral default mode network (vDMN): posterior cingulate cortex, precuneus, and angular and inferior parietal lobe; (4) right executive control network (rECN): right dorsolateral prefrontal cortex, posterior cingulate cortex; (5) salience network (SN): anterior insula, dorsal anterior cingulate cortex; (6) left executive control network (lECN): left dorsolateral prefrontal cortex, posterior cingulate cortex; (7) auditory network (AN): bilateral middle and superior temporal gyrus; (8) sensorimotor network (SMN): precentral gyrus and postcentral gyrus and supplementary motor area (SMA); and (9) dorsal default mode network (dDMN): medial superior frontal cortex and anterior cingulate cortex.

### 3.3. Altered RSNs in the DR Group

**Figure 2:**
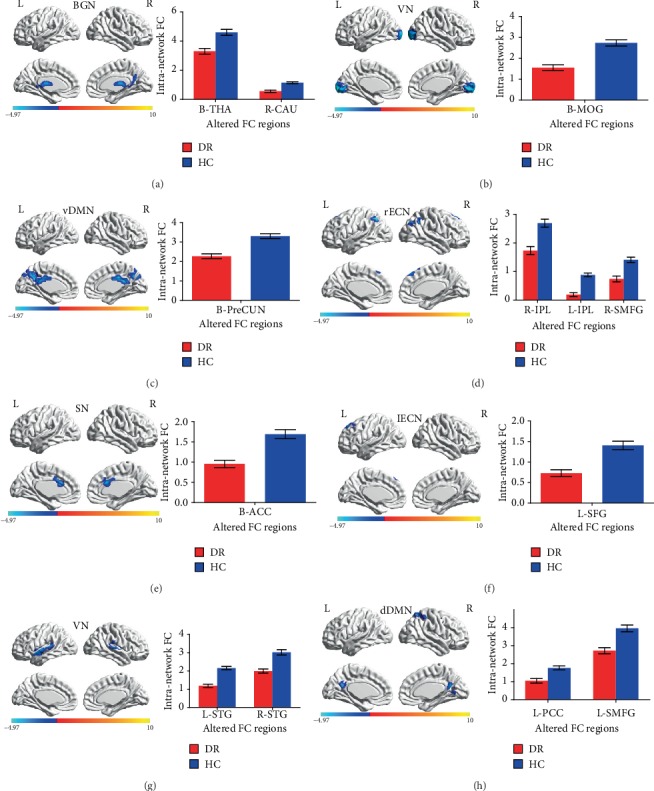
Brain regions with significant differences for eight RSNs in the DR group vs. the HC group (two-tailed, voxel-level *P* < 0.01; GRF correction, cluster-level *P* < 0.05). Cool colors indicated the decreased intranetwork functional connectivity FC in the DR group compared with the HC group, as shown by BrainNet Viewer. (a–h) correspond to different resting-state networks. BGN, VN, vDMN, rECN, SN, lECN, AN, and dDMN. Abbreviations: DR: diabetic retinopathy; HC: healthy control; BGN: basal ganglia network; VN: visual network; vDMN: ventral default mode network; rECN: right executive control network; SN: salience network; lECN: left executive control network; AN: auditory network; dDMN: dorsal default mode network; RSN: resting-state networks; THA: thalamus; CAU: caudate; MOG: middle occipital gyrus; PreCUN: precuneus; IPL: inferior parietal lobule; SMFG: superior medial frontal gyrus; ACC: anterior cingulate gyrus; SFG: superior frontal gyrus; STG: superior temporal gyrus; PCC: posterior cingulate gyrus; GRF: Gaussian random field; L: left; R: right; B: bilateral.

Significant decreased intranetwork FC within RSNs was identified in the DR group relative to the HC group (Figure 2 and Table 2). Compared with the HC group, the DR group showed decreased intranetwork FC in the bilateral thalamus and right caudate of the BGN (Figure 2(a)), the bilateral middle occipital gyrus of the VN (Figure 2(b)), the bilateral precuneus of the vDMN (Figure 2(c)), the right inferior parietal lobule and left inferior parietal lobule and right superior medial frontal gyrus of the rECN (Figure 2(d)), the bilateral anterior cingulate gyrus of the SN (Figure 2(e)), the left superior frontal gyrus of the lECN (Figure 2(f)), the left superior temporal gyrus and right superior temporal gyrus of the AN (Figure 2(g)), and the left posterior cingulate gyrus and left superior medial frontal gyrus of the dDMN (Figure 2(h)) (two-tailed, voxel-level *P* < 0.01; GRF correction, cluster-level *P* < 0.05).

### 3.4. FNC Analysis

**Figure 3:**
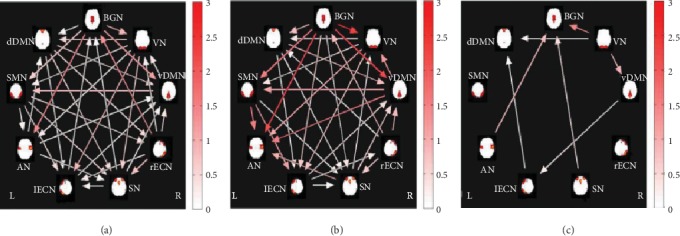
Images show group FNC of RSN obtained by using the one-sample *t*-test in patients with DR (a) and HC (b). Significant different FNCs of RSN between patients with DR and HC using two-sample *t*-test. (c) The color bars represent time lag (0–3 s). Arrows represented a significant correlation between RSNs (*P* < 0.05, uncorrected); FNC analysis showed the increased functional network connectivity between VN-BGN, VN-vDMN, VN-dDMN, vDMN-lECN, SN-BGN, lECN-dDMN, and AN-BGN in the DR group relative to the HC group. Abbreviations: DR: diabetic retinopathy; HC: healthy control; BGN: basal ganglia network; VN: visual network; vDMN: ventral default mode network; rECN: right executive control network; SN: salience network; lECN: left executive control network; AN: auditory network; dDMN: dorsal default mode network; FNC: functional network connectivity; RSN: resting-state networks; L: left; R: right.

Arrows represented a significant correlation between RSNs. FNC analysis showed the increased functional network connectivity between VN-BGN, VN-vDMN, VN-dDMN, vDMN-lECN, SN-BGN, lECN-dDMN, and AN-BGN in the DR group relative to the HC group (*P* < 0.05, uncorrected) (Figure 3).

### 3.5. Correlation Analysis

**Figure 4:**
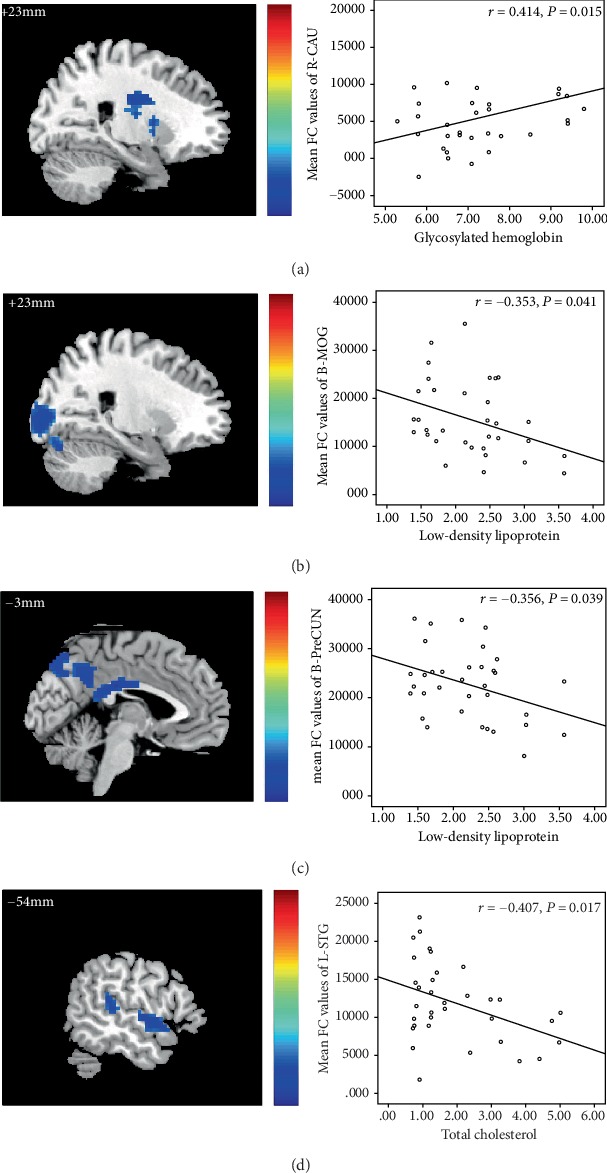
The HbA1c of DR patients showed a positive correlation with the intranetwork FC values of the right CAU (*r* = 0.414, *P* = 0.015) (a), and the low-density lipoprotein of DR patients showed a negative correlation with the intranetwork FC values of the bilateral MOG (*r* = −0.353, *P* = 0.041) (b). The low-density lipoprotein of DR patients showed a negative correlation with the intranetwork FC values of bilateral PreCUN (*r* = −0.356, *P* = 0.039) (c). The total cholesterol of DR patients showed a negative correlation with the intranetwork FC values of left STG (*r* = −0.407; *P* = 0.017) (d).

The HbA1c of DR patients showed a positive correlation with the intranetwork FC values of the right CAU (*r* = 0.414, *P* = 0.015) (Figure 4(a)), and the low-density lipoprotein of DR patients showed a negative correlation with the intranetwork FC values of the bilateral MOG (*r* = −0.353, *P* = 0.041) (Figure 4(b)). The low-density lipoprotein of DR patients showed a negative correlation with the intranetwork FC values of bilateral PreCUN (*r* = −0.356; *P* = 0.039) (Figure 4(c)). The total cholesterol of DR patients showed a negative correlation with the intranetwork FC values of left STG (*r* = −0.407; *P* = 0.017) (Figure 4(d)).

### 3.6. ROC Analysis for Discrimination

**Figure 5:**
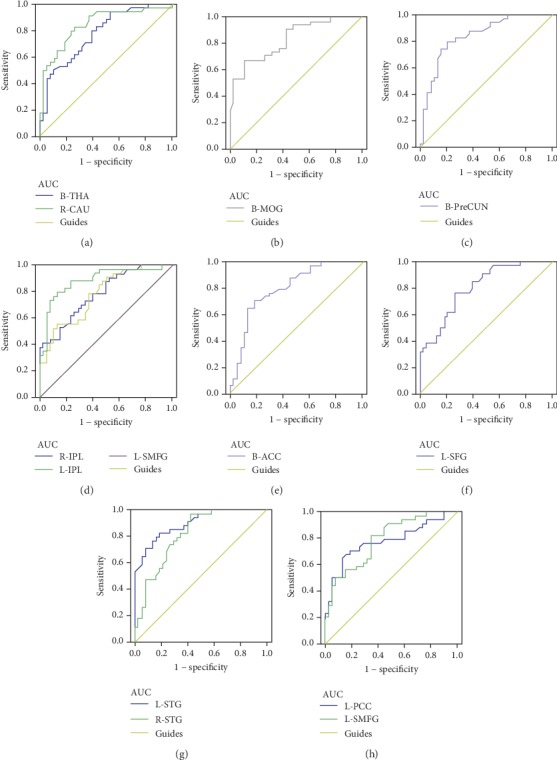
ROC curve analysis of the mean intranetwork FC and for altered brain regions. Note: ROC curve in intranetwork FC values: DR < HC, for B-THA, 0.776 (*P* < 0.001; 95% CI: 0.670–0.882); for R-CAU, 0.843 (*P* < 0.001; 95% CI: 0.749–0.937) (a); for B-MOG, 0.835 (*P* < 0.001; 95% CI: 0.744–0.926) (b); for B-PreCUN, 0.839 (*P* < 0.001; 95% CI: 0.747–0.932) (c); for R-IPL, 0.787 (*P* < 0.001; 95% CI: 0.684–0.889); for L-IPL, 0.887 (*P* < 0.001; 95% CI: 0.807–0.966); for L-SMFG, 0.781 (*P* < 0.001; 95% CI: 0.676–0.885) (d); for B-ACC, 0.801 (*P* < 0.001; 95% CI: 0.698–0.903) (e); for L-SFG, 0.804 (*P* < 0.001; 95% CI: 0.705–0.902) (f); for L-STG, 0.899 (*P* < 0.001; 95% CI: 0.831–0.967); for L-STG, 0.817 (*P* < 0.001; 95% CI: 0.720–0.913) (g); for L-PCC, 0.778 (*P* < 0.001; 95% CI: 0.666–0.889); and for L-SMFG, 0.791 (*P* < 0.001; 95% CI: 0.689–0.893) (h). Abbreviations: ROC: receiver operating characteristic; FC: functional connectivity; AUC: area under the curve; THA: thalamus; CAU: caudate; MOG: middle occipital gyrus; PreCUN: precuneus; IPL: inferior parietal lobule; SMFG: superior medial frontal gyrus; ACC: anterior cingulate gyrus; SFG: superior frontal gyrus; STG: superior temporal gyrus; PCC: posterior cingulate gyrus; GRF: Gaussian random field; L: left; R: right; B: bilateral.

The ROC curve in intranetwork FC values are the following: DR < HC, for bilateral THA, 0.776 (*P* < 0.001; 95% CI: 0.670-0.882); for right CAU, 0.843 (*P* < 0.001; 95% CI: 0.749-0.937) (Figure 5(a)); for bilateral MOG, 0.835 (*P* < 0.001; 95% CI: 0.744-0.926) (Figure 5(b)); for bilateral PreCUN, 0.839 (*P* < 0.001; 95% CI: 0.747-0.932) (Figure 5(c)); for right IPL, 0.787 (*P* < 0.001; 95% CI: 0.684-0.889); for left IPL, 0.887 (*P* < 0.001; 95% CI: 0.807-0.966); for left SMFG, 0.781 (*P* < 0.001; 95% CI: 0.676-0.885) (Figure 5(d)); for bilateral ACC, 0.801 (*P* < 0.001; 95% CI: 0.698-0.903) (Figure 5(e)); for left SFG, 0.804 (*P* < 0.001; 95% CI: 0.705-0.902) (Figure 5(f)); for left STG, 0.899 (*P* < 0.001; 95% CI: 0.831-0.967); for left STG, 0.817 (*P* < 0.001; 95% CI: 0.720-0.913) (Figure 5(g)); for left PCC, 0.778 (*P* < 0.001; 95% CI: 0.666-0.889); and for left SMFG, 0.791 (*P* < 0.001; 95% CI: 0.689-0.893) (Figure 5(h)).

## 4. Discussion

To the best of our knowledge, this is the first study to investigate whether the intranetwork FCs and FNCs of RSNs change in DR patients. Our study revealed that DR patients showed significantly decreased intranetwork FCs in both low-level perceptual (BGN, VN, and AN) and higher-order cognitive networks (vDMN, rECN, SN, lECN, and dDMN). Moreover, the DR group showed increased VN-BGN, VN-vDMN, VN-dDMN, vDMN-lECN, SN-BGN, lECN-dDMN, and AN-BGN FNCs, relative to the HC group. The decreased intranetwork FCs of RSNs were correlated with clinical variables in the DR group.

The VN is located in the occipital cortex up to the temporal-occipital junctions, which play an important role in processing visual information [39]. The main pathological changes in DR are retinal capillary nonperfusion, as well as vascular leakage and degeneration. In addition, DR causes retinal neurodegeneration [40]. Thus, visual loss is an important clinical manifestation in DR patients. Furthermore, DR patients exhibit abnormalities in the visual cortex. Ozsoy et al. demonstrated that DR patients showed decreased N-acetyl-aspartate/creatine and N-acetyl-aspartate/choline ratios in the visual cortex, relative to HCs [41]. Ferreira et al. found that decreased gray matter volume in the occipital lobe was detected in diabetic patients without retinopathy, relative to HCs [42]. Consistent with these findings, our results revealed that DR patients showed decreased intranetwork FC in the bilateral middle occipital gyrus of the VN. Moreover, Low-density lipoprotein in DR patients was negatively correlated with the intranetwork FC values of the bilateral MOG (*r* = −0.353, *P* = 0.041). Thus, we speculated that DR patients would exhibit an impaired VN and that the higher Low-density lipoprotein level would be closely correlated with abnormalities in the VN in DR patients.

The AN is located in the temporal lobe, which plays an important role in processing auditory information. There is growing evidence that impaired auditory function is present in diabetes patients [43–46]. In addition, several neuroimaging studies have revealed that diabetes patients exhibit temporal lobe atrophy. Willette et al. found that insulin resistance was correlated with medial temporal lobe atrophy and was related to cognitive deficits [47]. Northam et al. reported that type 1 diabetes mellitus patients showed decreased white matter in the left temporal lobe [48]. Chen et al. demonstrated that T2DM patients had gray matter atrophy in the temporal gyri, relative to HCs [49]. Consistent with these findings, our results revealed decreased intranetwork FCs in the left superior temporal gyrus and right superior temporal gyrus of the AN, implicating impaired auditory function in DR patients.

The BGN are activated for specific functions and circumstances, including movement control [50], associative learning [51], working memory [52], and emotion [53]. Previous studies demonstrated that the basal ganglia play a critical role in motor control [54, 55]. There are increasing reports that abnormalities in the BGN are present in central nervous system diseases, including Parkinson's disease [56], schizophrenia [57], and Alzheimer's disease [58]. Prior studies demonstrated that basal ganglia lesions occurred in diabetic nephropathy and diabetic uremia patients, indicating potential movement disorders in diabetes patients [59, 60]. Consistent with these findings, our results revealed that the DR group had decreased intranetwork FCs in the bilateral thalamus and right caudate of the BGN. Thus, these findings suggested that DR patients might exhibit movement control dysfunction.

The DMN is regarded as an endogenous neural network that shows consistently higher blood oxygenation level-dependent activity during rest; it plays an important role in self-referential thought and introspection [61]. The DMN consists of several brain regions including the medial prefrontal cortex, posterior cingulate cortex, inferior parietal cortex, and precuneus [62]. These regions are involved in various higher-cognition functions, such as memory, prospection, and self-processing [63, 64]. There is growing evidence that diabetes patients exhibit abnormal FC of the DMN, which is correlated with cognitive decline [65–67]. In addition, Chen et al. reported that T2DM patients had disrupted DMN organization, which was related to episodic memory in these patients [68]. Consistent with these findings, our results revealed that DR patients showed significantly decreased intranetwork FCs in the bilateral precuneus, left posterior cingulate gyrus, and left superior medial frontal gyrus of the DMN, which suggests cognitive decline in DR patients. Moreover, we found a negative association between the decreased intranetwork FC of the bilateral precuneus and low-density lipoprotein levels in DR patients. We presume that the lipid metabolism level might affect cognitive function in DR patients.

The ECN is involved in goal-directed selection of stimuli and responses, as well as cognitive control [69–71]. It consists of several brain regions, including the dorsolateral prefrontal cortex and posterior parietal cortex [72, 73]. Moran et al. reported that T2DM patients showed decreased gray matter volume in medial temporal, anterior cingulate, and medial frontal lobes, which were related to poor visuospatial construction, planning, and visual memory [74]. Bolo et al. reported that diabetic patients exhibited increased FC of the right anterior insula and prefrontal cortex within the executive control network during hypoglycemia [75]. In addition, neuroimaging studies revealed that T2DM patients showed impaired ECN relative to nondiabetic HCs [76]. Consistent with the findings of the prior studies, we found that DR patients showed decreased intranetwork FCs of the bilateral inferior parietal lobule, right superior medial frontal gyrus, and left superior frontal gyrus of the ECN. Our results suggest that decreased FCs within the ECN might reflect impaired executive control and cognitive control in DR patients.

The SN is involved in identifying the most relevant stimuli among several internal and external stimuli to guide behavior, which consists of the dorsal anterior cingulate and anterior insula [77]. The activation of SN has been detected during attentional, working memory, and response-selection paradigms [78], which are involved in switching between the ECN and DMN [72]. Cui et al. demonstrated that T2DM patients showed decreased degree centrality in the left lingual gyrus and increased centrality in the right insula and dorsal anterior cingulate cortex in the SN [79]. Our study demonstrated that DR patients had decreased intranetwork FC of the bilateral anterior cingulate gyrus of the SN. Thus, we speculate that DR patients might demonstrate impaired attentional and working memory function.

In our study, FNC analysis showed the increased VN-BGN, VN-vDMN, VN-dDMN, vDMN-lECN, SN-BGN, lECN-dDMN, and AN-BGN FNCs in the DR group, relative to the HC group. The SN, ECN, and DMN were the high-level cognitive networks. The activity within these three functionally connected networks depended on the task characteristics and complexity, as well as whether the task involved cognitive, emotional, sensory, or interoceptive stimuli [69, 80, 81]. A previous study demonstrated that interactions among the SN, ECN, and DMN are involved in working memory load [82]. Abnormal FCs among the ECN, DMN, and SN have been shown to contribute to cognitive decline in Alzheimer's disease patients [83]. Thus, our results suggest that DR patients might also exhibit cognitive decline. In addition, increased FC between the VN and DMN was observed in DR patients. Previous studies demonstrated that visual loss could cause changes in high-level cognitive networks [37]. Thus, we speculate that increased VN-vDMN and VN-dDMN FNC might reflect RSN compensation in DR patients with visual loss.

The ROC curve was conducted to assess the sensitivity of the intranetwork FCs of RSNs in discriminating between the two groups. Accuracy is perceived as excellent when AUC values are 0.7–0.9 discrimination between two groups. Our results demonstrated that the negative intranetwork FCs of RSNs showed a high degree of sensitivity and specificity to discriminate two groups (AUC values in 0.7-0.9). Specifically, the AUC for left IPL of the right executive control network (0.887) and for left STG of the auditory network (0.899) showed high sensitivity. Thus, the intranetwork FCs of these RSNs might be a potential biomarker for identifying neural mechanism dysfunction in DR patients.

Some limitations should be acknowledged in this study. First, the sample size of DR patients in our study was small, which may limit the generalizability of the findings. Second, the lack of psychological and cognitive tests prevented us from investigating the relationship between RSNs and neuropsychological characteristics in these DR patients. Additional studies should be performed to investigate these relationships. Third, RSN values based on blood oxygenation level-dependent signals would still be affected by physiological noise, such as cardiac and respiratory activity. In future studies, we plan to enlarge the sample size. Multimodal magnetic resonance imaging technologies will also be used to determine functional and morphological changes in DR patients.

## 5. Conclusions

In conclusion, our results revealed that DR patients had widespread deficits in both low-level perceptual and higher-order cognitive networks, which suggest potential impairments in visual, auditory, and cognitive functions in DR patients. Our results provide useful information to better understand the neural mechanisms that affect DR patients.

## Figures and Tables

**Table 1:** Demographics and visual measurements between two groups.

	DR group	HC group	*t* values	*P* values
Gender (male/female)	18/16	15/23	N/A	N/A
Age (years)	53.53 ± 8.67	48.63 ± 11.83	1.984	0.051
Handedness	34 R	38 R	N/A	N/A
Education (years)	11.91 ± 1.64	12.13 ± 1.59	-0.576	0.567
BMI (kg/m^2^)	23.91 ± 2.23	23.03 ± 1.92	1.801	0.076
BCVA-OD	0.49 ± 0.28	1.36 ± 0.15	-16.665	<0.001
BCVA-OS	0.43 ± 0.30	1.17 ± 0.21	-12.104	<0.001
HbA1c (%)	7.34 ± 1.34	N/A	N/A	N/A
Fasting blood glucose (mmol/L)	7.87 ± 2.54	N/A	N/A	N/A
Total cholesterol (mmol/L)	1.91 ± 1.38	N/A	N/A	N/A
Triglyceride (mmol/L)	3.69 ± 1.21	N/A	N/A	N/A
HDL cholesterol (mmol/L)	1.11 ± 0.28	N/A	N/A	N/A
LDL cholesterol (mmol/L)	2.22 ± 0.60	N/A	N/A	N/A

Note: *χ*^2^ test for sex (*n*). Independent *t* test for the other normally distributed continuous data (means ± SD). Abbreviations: DR: diabetic retinopathy; HC: healthy control; N/A: not applicable; BCVA: best corrected visual acuity; OD: oculus dexter; OS: oculus sinister; Hb: glycosylated hemoglobin; BMI: body mass index; HDL: high-density lipoprotein; LDL: low-density lipoprotein.

**Table 2:** Different intranetwork FCs of RSNs between two groups.

Condition	RSN	Brain regions	BA	Peak *t* scores	MNI coordinates (*x*, *y*, *z*)	Cluster size (voxels)
DR < HC	BGN	B-THA	—	-4.0477	3, -21, 12	121
		R-CAU	—	-4.9691	18, -12, 24	182
DR < HC	VN	B-MOG	17, 18	-5.4465	-15, -96, 0	1497
DR < HC	vDMN	B-PreCUN	—	-4.4778	3, -24, 30	562
DR < HC	rECN	R-IPL	40	-3.8525	45, -57, 57	155
		L-IPL	40	-5.6977	-45, -69, 48	181
		L-SMFG	8	-3.3675	9, 36, 51	96
DR < HC	SN	B-ACC	24	-4.1853	3, 21, 24	133
DR < HC	lECN	L-SFG	8	-3.7062	-15, 45, 42	78
DR < HC	AN	L-STG	42	-4.7819	-66, -30, 15	263
		R-STG	40	-4.3684	60, -24, 15	165
DR < HC	dDMN	L-PCC	—	-4.2416	3, -63, 3	94
		L-SMFG	8	-4.082	0, 39, 57	83

Note: the statistical threshold was set at the voxel level with *P* < 0.01 for multiple comparisons using Gaussian random field theory (voxel-level *P* < 0.01, GRF correction, cluster-level *P* < 0.05). *t* score represents the statistical value of peak voxel showing the differences in FC between the two groups. Abbreviations: DR: diabetic retinopathy; HC: healthy control; FC: functional connectivity; RSNs: resting-state networks; BA: Brodmann area; MNI: Montreal Neurologic Institute; BGN: basal ganglia network; VN: visual network; vDMN: ventral default mode network; rECN: right executive control network; SN: salience network; lECN: left executive control network; AN: auditory network; dDMN: dorsal default mode network; RSN: resting-state networks; THA: thalamus; CAU: caudate; MOG: middle occipital gyrus; PreCUN: precuneus; IPL: inferior parietal lobule; SMFG: superior medial frontal gyrus; ACC: anterior cingulate gyrus; SFG: superior frontal gyrus; STG: superior temporal gyrus; PCC: posterior cingulate gyrus; GRF: Gaussian random field; L: left; R: right; B: bilateral.

## Data Availability

The MRI data used to support the findings of this study are available from the corresponding author upon request.
